# Graph neural network based on brain inspired forward-forward mechanism for motor imagery classification in brain-computer interfaces

**DOI:** 10.3389/fnins.2024.1309594

**Published:** 2024-03-28

**Authors:** Qiwei Xue, Yuntao Song, Huapeng Wu, Yong Cheng, Hongtao Pan

**Affiliations:** ^1^Institute of Plasma Physics, Hefei Institutes of Physical Science, Chinese Academy of Sciences, Hefei, China; ^2^University of Science and Technology of China, Hefei, China; ^3^Mechanical Department, School of Energy Systems, Lappeenranta University of Technology (LUT), Lappeenranta, Finland

**Keywords:** brain computer interface (BCI), Electroencephalography (EEG), motor imagery (MI), forward-forward mechanism, Graph Convolutional Network (GCN)

## Abstract

**Introduction:**

Within the development of brain-computer interface (BCI) systems, it is crucial to consider the impact of brain network dynamics and neural signal transmission mechanisms on electroencephalogram-based motor imagery (MI-EEG) tasks. However, conventional deep learning (DL) methods cannot reflect the topological relationship among electrodes, thereby hindering the effective decoding of brain activity.

**Methods:**

Inspired by the concept of brain neuronal forward-forward (F-F) mechanism, a novel DL framework based on Graph Neural Network combined forward-forward mechanism (F-FGCN) is presented. F-FGCN framework aims to enhance EEG signal decoding performance by applying functional topological relationships and signal propagation mechanism. The fusion process involves converting the multi-channel EEG into a sequence of signals and constructing a network grounded on the Pearson correlation coeffcient, effectively representing the associations between channels. Our model initially pre-trains the Graph Convolutional Network (GCN), and fine-tunes the output layer to obtain the feature vector. Moreover, the F-F model is used for advanced feature extraction and classification.

**Results and discussion:**

Achievement of F-FGCN is assessed on the PhysioNet dataset for a four-class categorization, compared with various classical and state-of-the-art models. The learned features of the F-FGCN substantially amplify the performance of downstream classifiers, achieving the highest accuracy of 96.11% and 82.37% at the subject and group levels, respectively. Experimental results affirm the potency of FFGCN in enhancing EEG decoding performance, thus paving the way for BCI applications.

## 1 Introduction

Brain-Computer Interface (BCI) technology facilitates information exchange between the human brain and external devices, enabling information transmission bypassing the traditional nerve and muscle pathways (Hou et al., 2022b). By circumventing conventional neural pathways and muscle systems, BCI has successfully established themselves in diverse domains such as exoskeleton-assisted rehabilitation, fatigue monitoring, and process control in the industry (Huang et al., 2023; Liu et al., 2023; Zhang R. et al., 2023). A prominent subset of BCI, benefiting from advances in signal processing and deep learning (DL), is Electroencephalography (EEG) (Gao and Mao, 2021; Zhao et al., 2022; Li H. et al., 2023). EEG technology primarily aims to identify and categorize motor imagery (MI) signals, a vital aid for individuals with mobility impairments such as stroke victims. EEG's high accuracy, real-time response and cost-effectiveness distinguish it from other neuroimaging techniques like magnetoencephalography and functional magnetic resonance imaging (Huang et al., 2021; Mirchi et al., 2022; Tong et al., 2023).

Conventional algorithms for MI-EEG classification employ spatial decoding techniques, leveraging multichannel EEG data recorded from the scalp to identify motor intentions (Xu et al., 2021). In an endeavor to classify signals sourced from multi-channel MI-EEG, various methods have been proposed, effectively capturing their temporal, spectral and spatial characteristics (Tang et al., 2019; Wang and Cerf, 2022; Hamada et al., 2023; Li Y. et al., 2023). Given the rhythmic and non-linear nature of EEG signals, several feature extraction techniques leveraging wavelet modulation and fuzzy entropy have been proposed. Grosse (Grosse-Wentrup and Buss, 2008) introduced a methodology that incorporates common spatial pattern (CSP) for spatial filtering and reducing dimensionality, which is supplemented with the filter bank technique to divide the spatially refined signals into multiple frequency sub-bands. On a similar note, Malan and Sharma (2022) developed a filter bank based on dual-tree complex wavelet transform to separate EEG signals into sub-bands. Once the EEG signals have been segmented into these sub-bands, spatial characteristics are derived from each sub-band through the CSP and subsequently refined employing a supervised learning framework. A multi-layer twin support vector machine leveraging phase space and wavelet transform is presented by Fei and Chu (2022). Despite their potential, these methodologies overlook the topological relationships among electrodes, necessitating further optimization to improve MI classification accuracy.

Recognizing the growing emphasis in neuroscience on brain network dynamics and neural signal propagation mechanism, Graph Convolutional Network (GCN) have been introduced to decode EEG signals (Wang et al., 2021; Du G. et al., 2022; Gao et al., 2022). Then Kipf and Welling (2016) combined graph theory and DL to capture the relationship between nodes. Coincidentally, the forward-forward (F-F) mechanism, a groundbreaking concept in neurotransmission introduced by Hinton (2022), is garnering attention. This mechanism provides an efficient method to process sequential data in neural networks without the need to store neural activities or pause for error propagation. Our study aims to integrate F-F mechanism with GCN for EEG-based BCI, proposing a significant advance in motor imagery classification.

In research, we put forward an innovative F-FGCN framework for MI classification. The salient contributions of our research are as follows:

1. A novel EEG classification model for four-class MI intentions called F-FGCN is presented, driven by brain network dynamics and neural signal propagation mechanism, incorporates brain-inspired F-F mechanism and cooperates with the functional topological relationships of EEG electrodes.
2. F-FGCN utilizes both pre-training and fine-tuning phases in GCN. By leveraging the pre-training process of the GCN, it effectively recognizes the relationships between multichannel EEG signals from the subjects, thus significantly enhancing the performance and robustness of our approach.
3. F-FGCN motivated by medical domain knowledge, alters the typical model where the neurons explicitly propagate error derivatives or stores neural activities for a subsequent backward pass. We replace backpropagation with F-F mechanism, generating a hybrid EEG feature by merging the feature with a mask. The creation of negative data involves the generation of a mask characterized by vast regions containing binary values of ones and zeros, using two consecutive forward passes to iterate over the parameters of positive and negative data.
4. Our model is benchmarked against a range of traditional and contemporary models through comprehensive experimental comparisons. Experimental results on the PhysioNet demonstrate that the proposed method achieved the highest accuracy of 93.06% and 88.57% at the subject and group level, respectively.

This paper is organized in the following manner: Section I outlines the BCI development and contributions of our research. Section 2 presents a detailed review of relevant background knowledge and related works. The structure of our model is delineated in Section 3. The results from our model and comparison tests are elaborated in Section 4. The discussion takes place in Section 5. The paper wraps up with concluding remarks and a glimpse into future work in Section 6.

## 2 Related work

BCI systems pivot around feature extraction and classification for executing MI tasks. Recent studies have largely focused on the feature extraction and classification of EEG signals within the DL framework.

### 2.1 Feature extraction

In recent times, DL has achieved remarkable performance across various fields due to its ability to extract underlying features from signals, thus mitigating the necessity for manual feature engineering. The Convolutional Neural Network (CNN) has found extensive usage in classifying Euclidean-structured signals, credited to its capability to learn informative features through local receptive fields (Du Y. et al., 2022; Mughal et al., 2022; de Oliveira and Rodrigues, 2023).

Tang et al. (2023a) developed a Multi-Scale Hybrid CNN to isolate temporal and spatial EEG signal attributes. Advanced temporal features were captured through the utilization of one-dimensional convolution, yielding impressive accuracies of 85.25% and 84.86% on BCI competition IV datasets 2b and 2a, respectively. Similarly, Jia et al. (2023) engineered a multi-branch CNN module for learning spectral-temporal domain characteristics. By incorporating a channel attention mechanism, more discriminative features were extracted, leading to 74% average accuracy on four class data. Moreover, Hu et al. (2023) employed band common spatial pattern coupled with duplex mean-shift clustering to extract diverse features across temporal, spectral and spatial domains. By combining these with CNN, features from different domains were consolidated, significantly improving the classification results.

However, conventional CNN encounters challenges in processing non-Euclidean structured data, primarily due to the inherent inability of discrete convolution to preserve translation invariance on non-Euclidean. To overcome this issue, GCN can process graph-structured signals and extract features from non-Euclidean data, taking into account the relationship properties between nodes. When coupled with functional topological relationships between electrodes, GCN can enhance the decoding efficiency of EEG tasks.

The concept of spatial GCN was initially introduced by studies (Song et al., 2020; Yang et al., 2020; Bui et al., 2022). In a noteworthy development, Liang et al. (2022) designed a GCN model for channel classification, treating all EEG channels as graph nodes, thus transforming channel selection into a graph node classification problem. In a different approach, Hou et al. (2022a) formulated a model that leveraged bidirectional LSTM with attention mechanism. By coordinating the GCN with the topological structure of features extracted from the total data, they significantly improved decoding performance. Similarly, Ye et al. (2022) introduced a hierarchical dynamic GCN that investigates dynamic spatial information at multiple levels across EEG channels. Their method consistently delivered superior results in extensive tests on the SJTU emotion EEG dataset.

### 2.2 Feature classification

The superiority of DL in signal processing has prompted researchers to adopt end-to-end algorithms based on the backpropagation mechanism for classification (Wang et al., 2020; Tang et al., 2023b; Zhang H. et al., 2023; Zhang J. et al., 2023). A multitude of models have been devised which transfigure unprocessed EEG signals into spatial-spectral-temporal forms for categorization, including CNN (Hossain et al., 2023), ANN (Subasi, 2005), and EEGNET (Lawhern et al., 2018).

Xiao et al. (2022) converted unprocessed EEG data into a 4D representation encompassing spatial, spectral, and temporal dimensions. Their method enhanced by spectral and spatial attention mechanisms, allowed to attribute distinct weights to various brain regions and frequency bands in a selective manner. On the other hand, Schirrmeister et al. (2017) developed three distinct CNN architectures, namely ShallowNet (Hu et al., 2021), DeepNet (Wang H. et al., 2022), and HybridNet (Dai, 2019), which served to decode MI-EEG from its raw EEG counterparts.

Wang Q. et al. (2022) put forth Anes-MetaNet, a model that applies CNN to extract power spectrum features and incorporates LSTM-based temporal to identify temporal dependencies. In parallel, Akmal (2022) employed tensor-based Canonical/Polyadiac Weighted-Optimization alongside ANN for the purpose of both rectifying missing data and performing classification tasks. Capitalizing on the transformer architecture strengths and the inherent spatial-temporal traits of EEG signals, Xie et al. (2022) developed transformer-centric models intended for classifying motor imagery EEG signals using the PhysioNet dataset. When implemented on 3s EEG data, this model exhibited remarkable classification accuracies, achieving 83.31%, 74.44%, and 64.22% for defferent MI classification tasks. Further, an unique graph sequence neural network was presented by Cai et al. (2022) to precisely decode motor imagery patterns from EEG data even amidst environmental distractions. Lastly, Umrani and Harshavardhanan (2022) harnessed ANN for anxiety detection, trained through their bespoke trace and forage optimization algorithm, which merges characteristics from rescue search agents and finches to enhance detection efficacy.

Nevertheless, most of the current DL methods heavily rely on backpropagation. These methods require a comprehensive understanding of computations performed during the forward pass, which can be a daunting task when the exact details of the forward computation are not available. The F-F mechanism enables learning by streaming sequential data through a neural network without the need to retain neural activities or interruption for error backpropagation. Consequently, the application of GCN based on the F-F mechanism for MI classification presents a novel approach.

## 3 Methods

**Figure 1:**
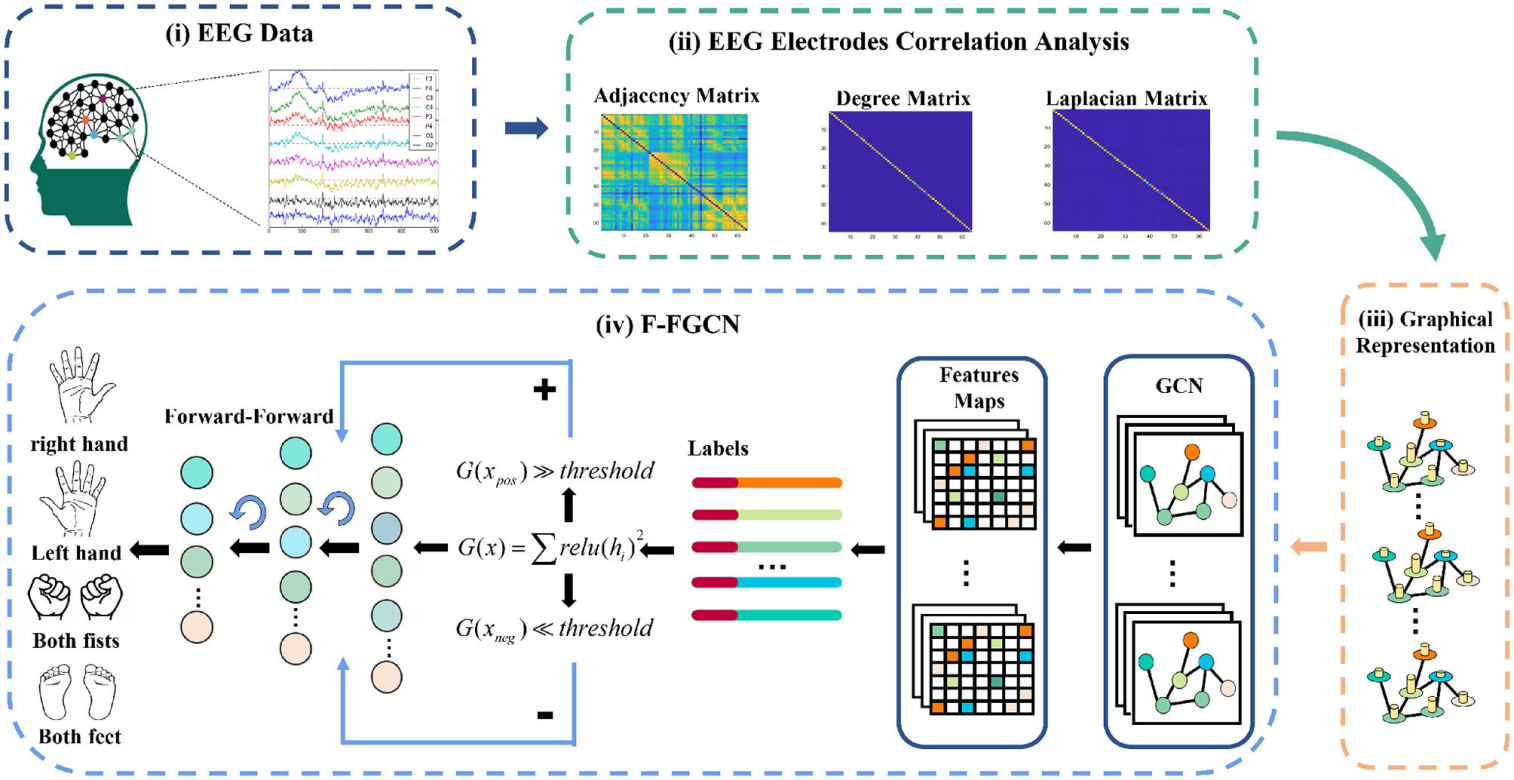
The system framework encompasses: (i) the collection of raw EEG signals, (ii) conducting correlation analysis for graph weights and degrees, demonstrated through the adjacency matrix, PCC matrix degree, and graph Laplacian, (iii) representing the international 10-10 EEG system in a graph, and (iv) integrating a new DL structure of the F-FGCN.

In this section, we thoroughly detail each block, and the overall framework. Firstly, we provide an explanation for the representation of EEG data in the Graph representation block, which forms the input for our proposed F-FGCN. Next, we delve into the structural components of the GCN block. Lastly, we leverage the F-F mechanism block to further extract EEG features for classification. Figure 1 provides a visual representation of the F-FGCN framework, consisting of three main blocks: the Graph representation block, the GCN block, and the F-F mechanism block.

### 3.1 Graph representation block

GCN is an appropriate choice to non-Euclidean data for its advantage for capturing topological structure information. EEG signals are naturally considered data with a graph structure. Electrodes, which collect EEG signals, are arranged on the surface of the brain following the guidelines of the 10-10 system.

An undirected weighted graph is symbolized as *G* = {*V, E*, **A**}, wherein *V* = {*v*_1_, *v*_2_, ..., *v*_*n*_} signifies the ensemble of *n* nodes. The set of *e* edges is represented by *ei* ∈ *E*. The weighted adjacency matrix, denoted as **A** ∈ *R*^*N*×*N*^, indicaed the linkages between any pair of nodes.

The Laplacian matrix of the graph *G* is defined as **L**, which can be written as Equation (1):


(1) $$\begin{array}{l} \text{L}=\text{D}-\text{A} \end{array}$$

where ${\text{D}}_{i,i}=\underset{j}{\sum }{\text{A}}_{i,j}$ is the degree matrix of the graph *G*.

The symmetric normalized graph Laplacian, **L**, is defined as Equation (2):


(2) $$\begin{array}{l} {\text{L}}_{normal}=\text{I}-{\text{D}}^{-1/2}\text{A}{\text{D}}^{-1/2} \end{array}$$

where **I** is the identify matrix. Normalized graph Laplacian **L**_*normal*_ represents the correlations between nodes.

For the purpose of illustrating a graph's degree matrix, we undertake the scale in graph weights, disregarding the direction of correlations. Consequently, the absolute value of the Pearson correlation coefficient (PCC) matrix is adopted. The PCC matrix is utilized to represent each electrode as a node in the graph, with edge weights determined by the correlations observed among the time-series signals in Equation (3):


(3) $$w_{i,j}=\left|\frac{\sum_{t=1}^{T}\left(x_{i}^{t}-{\bar{x}}_{i}\right)\left(x_{j}^{t}-{\bar{x}}_{j}\right)}{\sqrt{\sum_{t=1}^{T}{\left(x_{i}^{t}-{\bar{x}}_{i}\right)}^{2}}\sqrt{\sum_{t=1}^{T}{\left(x_{j}^{t}-{\bar{x}}_{j}\right)}^{2}}}\right|$$

where **x_i_**, **x_j_** are the signal vectors from nodes *v*_*i*_ and *v*_*j*_, and *T* is the total number of samples. **w_i, j_** ∈ [0, 1] can quantify the relationship between two channels and assess the strength of their correlation. A higher **w_i, j_** value indicates a stronger correlation between the channels.

### 3.2 GCN block

Spectral graph theory is rooted in the study of the structural attributes of graph data. The graph filter and graph convolution have been constructed using the Laplacian matrix. Signals on the nodes of the graph can be expressed as $f={\left[f_{1},f_{2},\ldots ,f_{n}\right]}^{⊤}$, and *f* ∈ ℝ^*n*^, where *f*_*i*_ is the value at the *ith* node. With the eigendecomposition of the graph Laplacian matrix **L** = **UΛU**^*T*^, the eigenvectors matrix $\text{U}=\left[{\text{u}}_{0},{\text{u}}_{1},...,{\text{u}}_{n\times n-1}\right]\in {\mathbb{R}}^{n\times n}$ is obtained, where $\text{Λ}=\text{diag}\left({\text{λ}}_{0},{\text{λ}}_{1},...,{\text{λ}}_{n-1}\right)\in {\mathbb{R}}^{n\times n}$ is a diagonal matrix with corresponding eigenvalues

The Graph Fourier Transform (GFT) for the initial signal **x** on the graph is expressed as Equation (4):


(4) $$\begin{array}{l} \hat{x}={\text{U}}^{T}\text{x} \end{array}$$

The inverse GFT is Equation (5):


(5) $$\begin{array}{l} \text{x}=\text{U}\hat{\text{x}} \end{array}$$

Per the convolution theorem, a convolution involving two signals can be transformed into a point-wise multiplication in the Fourier domain.

In accordance with the convolution theorem, considering a signal **x** as input and another signal **g** acting as a filter, the graph convolution **G** can be expressed as Equation (6):


(6) $$x{*}_{G}g=U((U^{T}x)⊙(U^{T}g))$$

In this context, ⊙ signifies the elementwise Hadamard product and **g** ∈ ℝ^*N*^ operates as a convolutional filter. Moreover, **g** is nonparametric and denoted as **g**_**θ**_(**Λ**) = diag(**θ**), with **θ** ∈ ℝ^*N*^ acting as the vector of Fourier coefficients. The convolution operation carried out in the GCN is as followed Equation (7):


(7) $$y=g_{\theta }(L)x=g_{\theta }(U\Lambda U^{T})x=Ug_{\theta }(\Lambda )U^{T}x$$

The differentiation in spectral graph convolution is primarily due to the choice of filter **g**_θ_. Owing to the fact that a nonparametric filter is not spatially localized and bears substantial computational complexity, we resort to polynomial approximation to resolve this matter. Chebyshev polynomials are commonly employed for filter approximation. Consequently, **g**_θ_ is parameterized as a truncated expansion in the following manner Equation (8):


(8) $$g_{\theta }\left(\Lambda \right)=\sum_{k=0}^{K}\theta_{k}T_{k}(\tilde{\Lambda })$$

In this equation, **θ** ∈ ℝ^*K*^ represents a set of Chebyshev coefficients, ${\text{T}}_{k}\left(\tilde{\text{Λ}}\right)\in {\mathbb{R}}^{K}$ is the *k*th-order Chebyshev polynomial assessed at ${\tilde{\text{Λ}}=2\Lambda /\Lambda }_{max}-{\text{I}}_{N}$, and **I**_*N*_ stands as a diagonal matrix with scaled eigenvalues. Then, the signal **x** is convolved by the defined filter **g**_θ_ as follows Equation (9):


(9) $$\begin{array}{l} \text{x}{*}_{\text{G}}{\text{g}}_{\theta }=\text{U}\overset{K-1}{\underset{k=0}{\sum }}\theta_{k}{\text{T}}_{k}\left(\tilde{\text{Λ}}\right){\text{U}}^{T}\text{x}=\overset{K-1}{\underset{k=0}{\sum }}\theta_{k}{\text{T}}_{k}\left(\tilde{\text{L}}\right)\text{x}. \end{array}$$

${\text{T}}_{k}\left(\tilde{\text{L}}\right)$ denotes the *k*th order Chebyshev polynomial evaluated at the rescaled Laplacian $\tilde{L}=2L/\Lambda_{max}-I_{N}$. Here, ${\bar{\text{x}}}_{k}={\text{T}}_{k}\left(\tilde{\text{L}}\right)\text{x}\in {\mathbb{R}}^{N}$, and a recursive relationship is employed to compute ${\bar{\text{x}}}_{k}$, such as ${\bar{\text{x}}}_{k}=\text{2}\tilde{\text{L}}{\bar{\text{x}}}_{k-1}-{\bar{\text{x}}}_{k-2}$ with initial conditions $\bar{{\text{x}}_{0}=x}$ and ${\bar{\text{x}}}_{1}=\tilde{\text{L}}x$.

The application of Chebyshev polynomial for the approximation of convolutional filters is advantageous since it lessens the demand for calculations related to graph Fourier basis. This subsequently leads to a decrease in computational complexity from *O*(*N*^2^) to a much more feasible *O*(*KN*).

### 3.3 Forward-forward block

Inspired by Boltzmann machines and Noise Contrastive Estimation, F-F provides speedy multi-layer learning, functioning even when forward computation specifics are obscured. F-F implements two alike forward passes instead of backpropagation's bidirectional passes, using dissimilar data and opposing goals. The positive pass uses authentic data to amplify the goodness of each hidden layer. The positive pass uses authentic data to amplify the goodness n of each hidden layer. In contrast, the F-F also includes another forward pass, which uses negative data generated by the network itself to adjust the weights in a manner that decreases the goodness measure in each hidden layer. The two forward passes replace the traditional forward and backward passes in backpropagation. Instead of calculating error gradients and propagating them back through the network as in backpropagation, the FF algorithm uses these two types of forward passes to adjust weights directly based on the goodness measure.

In experiments, only directions are used for subsequent layer transmission for several reasons. Firstly, to prevent the escalation of values, which could destabilize the network, especially in deep architectures. Secondly, focusing on the orientation rather than the magnitude encourages the network to discriminate features based on patterns of neuron activations. The activity vectors are normalized before entering the next layer to accelerate training convergence, prevent vanishing or exploding gradients, enhance generalization, mitigate overfitting, and maintain numerical stability, ensuring consistent learning progress.

The journey of an input vector *x* through the F-F commences with its propagation through the network to yield the hidden layer's output, *h*, articulated as Equation (10):


(10) $$\begin{array}{l} h=model\left(x\right) \end{array}$$

This step marks the genesis of the model's response to the input data. Central to the F-F is the concept of goodness, a metric that quantifies the hidden layer's reaction to input data. It's computed as the cumulative sum of the squares of the hidden units' activities within the layer. Mathematically, the current goodness *S*_*L*_ of a layer with output *h* is represented as Equation (11):


(11) $$\begin{array}{l} S_{L}=\overset{n}{\underset{j=1}{\sum }}h_{j}^{2} \end{array}$$

To navigate toward the desired state of the network, a target goodness level S^*^ is pre-established. The learning rate α is pivotal for steering the weight adjustments in alignment with this target. It's calculated using the formula Equation (12):


(12) $$\begin{array}{l} \alpha =\frac{S^{*}-1}{S_{L}} \end{array}$$

The initial hidden layer activity vector, with both length and orientation uses the former for defining layer specific goodness, and the latter for subsequent layer transmission. F-F utilizes real and corrupted data vectors that merge labels with the feature. The incoming weight increments for hidden neuron *j* are given by Equation (13):


(13) $$\begin{array}{l} \Delta {\text{w}}_{j}=2\epsilon \frac{\partial \log\left(p\right)}{\partial \underset{j}{\sum }y_{j}^{2}}y_{j}\text{x} \end{array}$$

After updating weights, the neuron *j*'s activity change equals Δ**w**_*j*_*x*, depending solely on *y*_*j*_, causing proportional changes in hidden activities without affecting the vector orientation.

An integral step involves discerning the input vector's probability of being positive, achieved by applying the logistic function σ to the goodness less a threshold θ in Equation (14):


(14) $$\begin{array}{l} p\left(\text{positive}\right)=\sigma \left(\sum_{j}h_{j}^{2}-\theta \right) \end{array}$$

Each layer's performance is gauged using a loss function designed to bifurcate the layer mean activity around a tunable threshold value θ. The function is Equation (15):


(15) $$\begin{array}{l} \mathrm{Loss}=\mathrm{mean}\left(\log\left(1+e^{\theta -\sum_{j=1}^{n}h_{j,P}^{2}}\right)+\log\left(1+e^{\sum_{j=1}^{n}h_{j,N}^{2}-\theta }\right)\right) \end{array}$$

*h*_*j, P*_ represents the activity of the *j* hidden unit for the positive sample, and *h*_*j, N*_ represents the activity for the negative sample.

**Figure 2:**
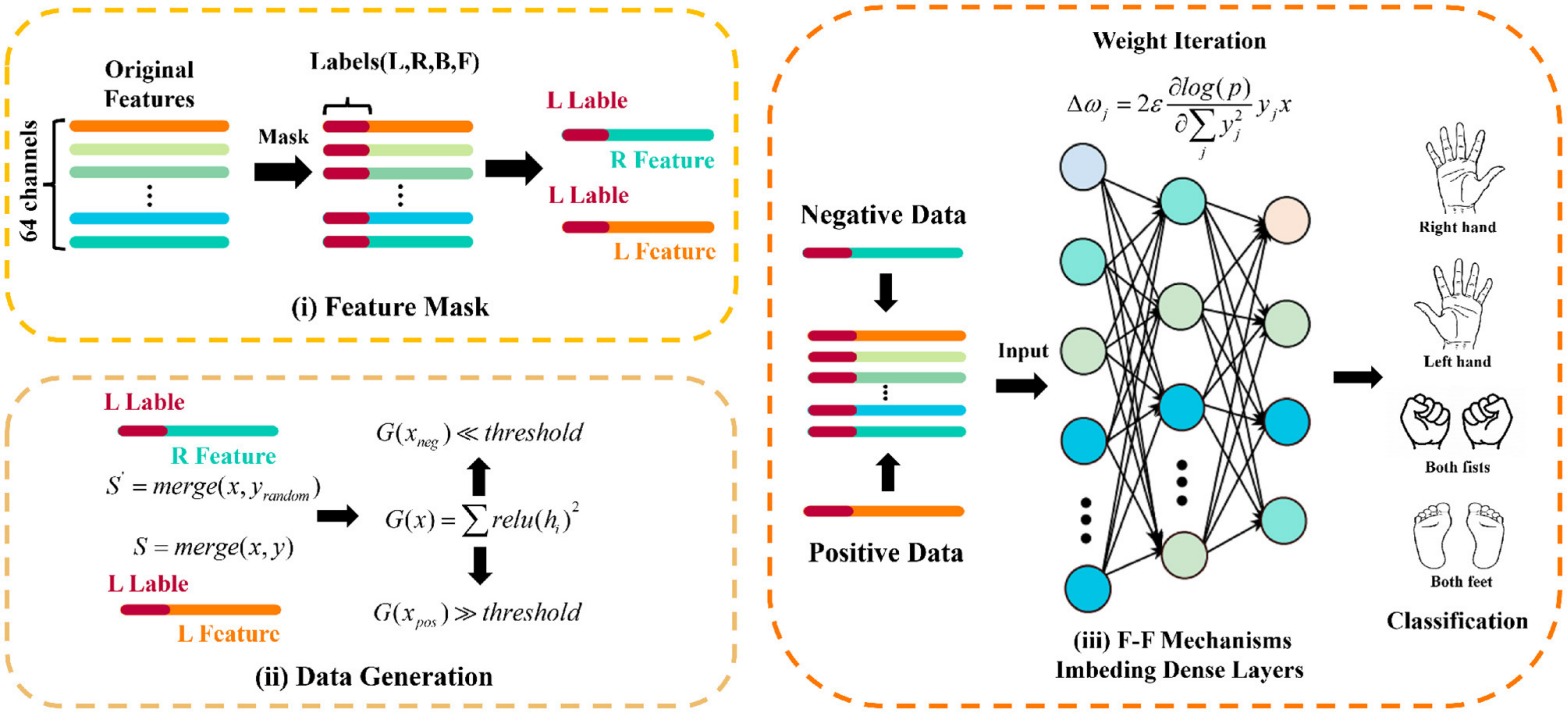
F-F block is composed of (i) Mask the features, (ii) Generate training data, and (iii) Training by F-F mechanism imbeding dense layers.

The iterative optimization process entails utilizing the normalized outputs of positive and negative data from preceding layers as inputs for successive layers. The process perpetuates until the loss value attains saturation, a hallmark of network convergence and its adeptness at differentiating between positive and negative data. The specific implementation of F-F block is shown in the Figure 2.

## 4 Experiments

### 4.1 Dataset and evaluation approaches

**Figure 3:**
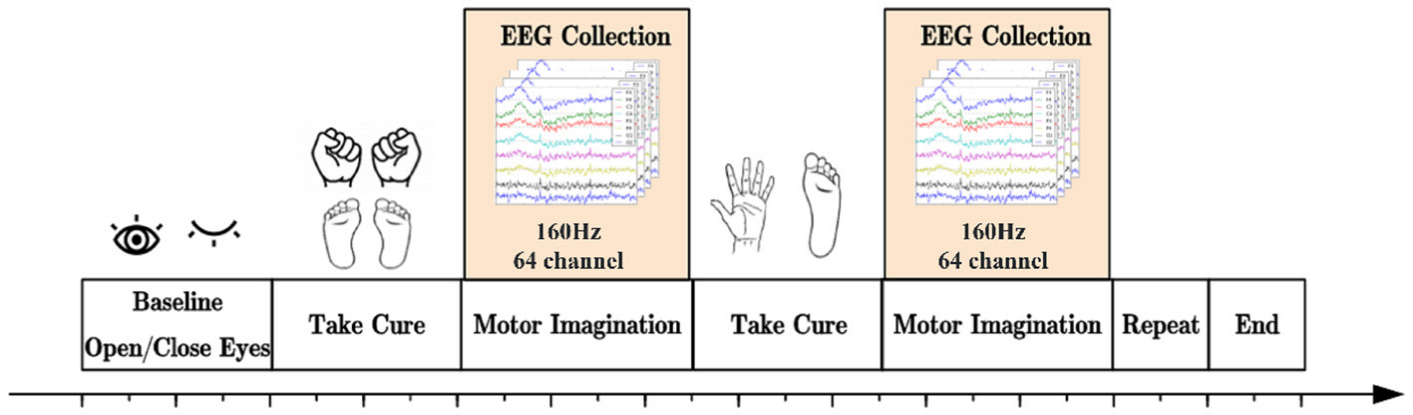
Experimental paradigm for the PhysioNet Dataset.

PhysioNet Dataset: This dataset comprises more than 1,500 EEG recordings from 109 participants, collected using 64 electrodes following the 10-10 system. It encompasses four distinct motor imagery tasks: L (left fist), R (right fist), B (both fists), and F (both feet), as depicted in Figure 3. Each subject performed 84 trials split into 3 runs of 7 trials in 4 tasks.

BCI Competition III 3a: This dataset focuses on cued motor imagery tasks with a multi-class approach, encompassing four distinct classes: left hand, right hand, foot, and tongue movements. The recorded data is extensive, with EEG readings taken from 60 channels, providing a comprehensive array of brain activity signals. Each of the four classes is well-represented, with 60 trials per class, offering a robust dataset for analysis and application in brain-computer interface research and development.

The effectiveness of our models is primarily evaluated on the basis of accuracy, determined by the Equation (16):


(16) $$\begin{array}{l} ACC=\frac{TP+TN}{TP+TN+FP+FN} \end{array}$$

Here, *TP* corresponds to true positives, *FP* refers to false positives, *FN* stands for false negatives, and *TN* indicates true negatives.

### 4.2 Training procedure

The model is trained using the TensorFlow framework, with training over 300 epochs harnessing the computational power of two Nvidia GTX 3090 GPU 24 GB of memory. Detailed parameters are outlined in Table 1.

**Table 1:** Training parameters.

**Model setup**	**Setting**
Framework	TensorFlow
Batch size	64
Learning rate	0.03
Optimizer	Adam
Metrics	Accuracy
Loss	Categorical cross-entropy
F-F layers	(512,256,256,4)
Device	2*Nvidia GTX 3090 24 GB

**Figure 4:**
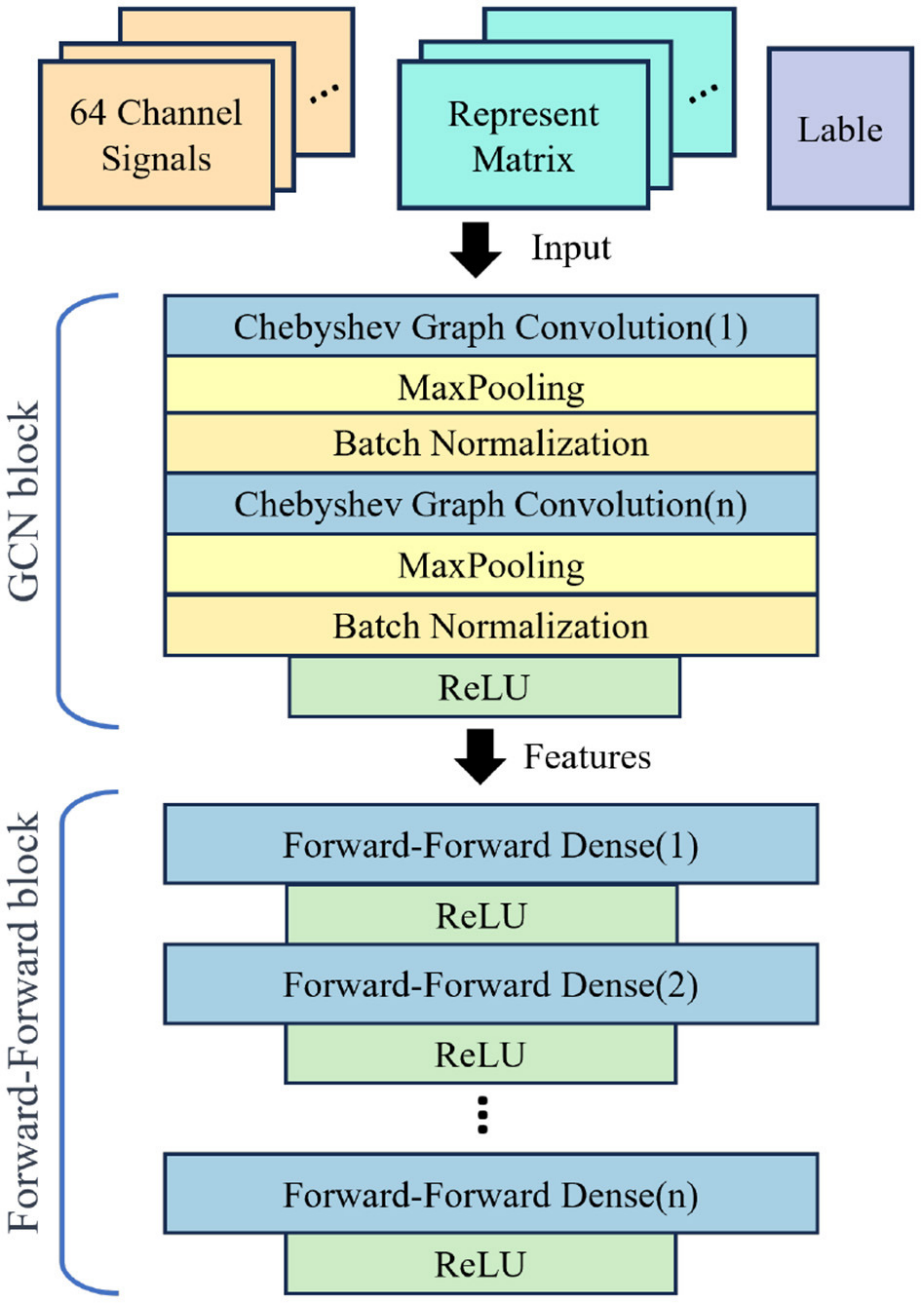
Architecture of F-FGCN. It consists of Chebyshev Graph Convolution, MaxPooling, and Forward-Forward Dense. Incorporating dense layers featuring Forward-Forward mechanism, which behind the GCN for enhanced feature extraction and categorization.

These specific hyperparameters are meticulously chosen after a series of comparative experiments, ensuring an optimal balance between performance and generalization of the models. Our experiments utilize a GCN model equipped with four graph convolution layers, with Chebyshev polynomial serving as the convolution kernel for GCN. The chosen structure for the GCN is depicted in Figure 4.

Detailed aspects of the model are provided in Table 2, “Maps” refers to the count of neurons present in each layer, “Size” signifies the dimension of the input feature for every corresponding layer, “*N*” is representative of the quantity of node features, “*K*” corresponds to the order of the Chebyshev Polynomial, and “*O*” denotes the classification category count. In the devised model, we incorporate dense layers featuring F-F mechanism, which is behind the GCN for enhanced feature extraction and categorization. The efficacy of this hierarchical design forms the key to our experimental analysis.

**Table 2:** Implementation details of GCN.

**Type**	**Maps**	**Size**	**Polynomial orders**	**Activation**	**Weights**	**Bias**
Input	—	*N*	—	—	—	—
C1	F1	*N*	K	ReLU	*N*×F1 × K	V × F1
Max-pooling	F1	*N*	—	—	—	—
C2	F2	*N*/2	K	ReLU	*N*/2 × F2 × K	V × F2
Max-pooling	F2	*N*/2	—	—	—	—
C3	F3	*N*/4	K	ReLU	*N*/4 × F3 × K	V × F3
Max-pooling	F3	*N*/4	—	—	—	—
C4	F4	*N*/8	K	ReLU	*N*/8 × F4 × K	V × F4
Max-pooling	F4	*N*/8	—	—	—	—
Output	—	O	—	ReLU	—	—

### 4.3 Experimental results

The performance of F-FGCN is benchmarked against both traditional and SOTA models. The data is randomly shuffled, creating datasets with identical data but in different orders. Subsequently, the data is partitioned into training, validation, and testing sets with a ratio of 7:2:1, respectively.

**Figure 5:**
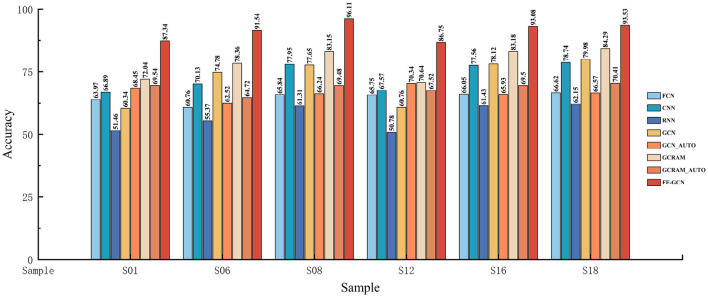
Bar plots of the accuracy comparing our model to the classical model.

We conducted cross-individual trials on the PhysioNet dataset utilizing our proposed network structure to evaluate the adaptability of F-FGCN to individual subjects. We select six subjects at random. As illustrated in Figure 5, F-FGCN demonstrated strong competitiveness, garnering an average classification accuracy of 89.39% across the six subjects.

**Figure 6:**
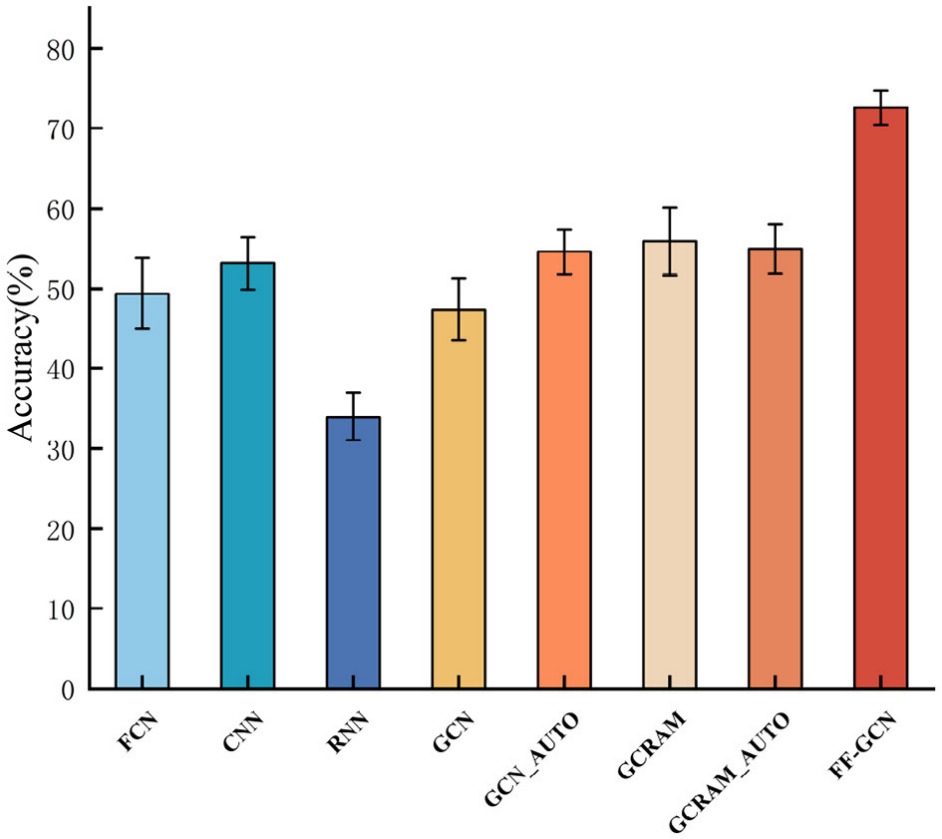
Plots with error bars comparing our model to the classical models.

In the context of the experiment, the convolution kernel sizes of the other networks are aligned with that of F-FGCN, with identical hyperparameters applied as provided in the experimental method section. Figure 6 exhibits the comparative results.

The precision of our model is juxtaposed with the results achieved by traditional models, including FCN, CNN, RNN, GCN, GCN_AUTO, GCRAM, and GCRAM_AUTO in Figure 6. Notably, GCN_AUTO and GCRAM_AUTO amalgamate a GCN and an autoencoder block to capture graph structures by reconstructing node-wise transformations from original and transformed graph features.

**Figure 7:**
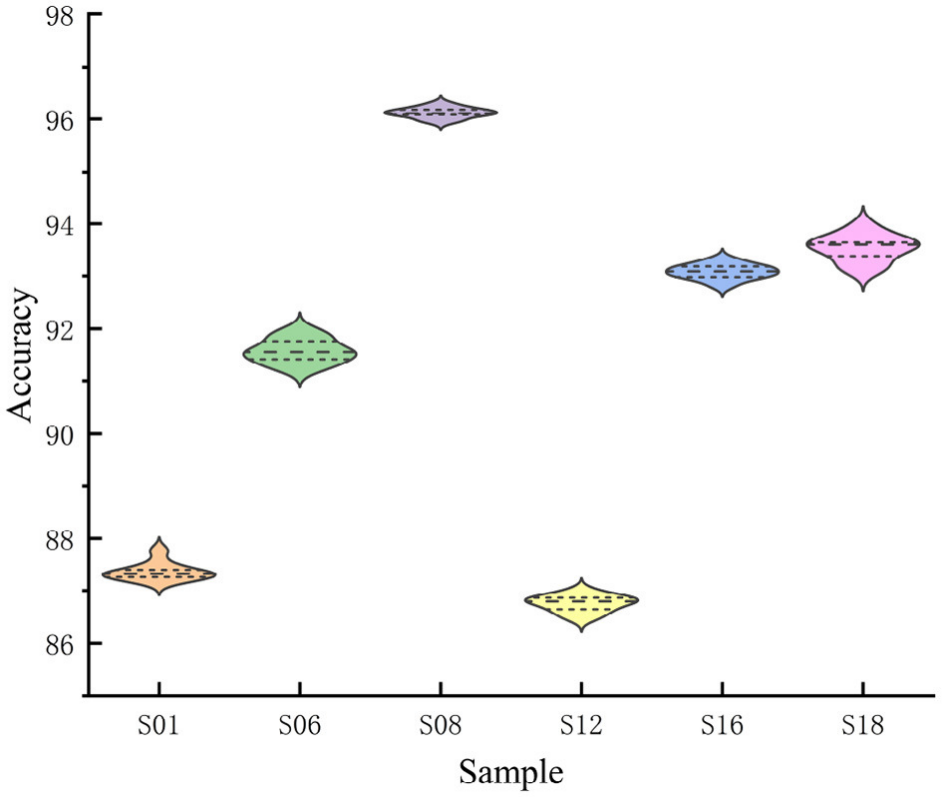
The violin plot illustrates the accuracy for six subjects, with the horizontal line symbolizing the average value, and every solid diamond signifies the classification precision for each specific subject.

Classification accuracy for the six subjects is presented in violin plots in Figure 7. The mean is represented by a horizontal line, while a solid diamond shows the classification accuracy distribution for each test. The kernel density representation outside the violin signifies a greater distribution probability surrounding more extensive graph regions. The F-FGCN model demonstrated commendable stability across different individual tests. However, the classification accuracy slightly reduces as the S12 dataset appears to be scattered.

Table 3 presents the classification results of recent SOTA approaches on the PhysioNet Dataset, with F-FGCN consistently outperforming other methods. F-FGCN achieves the average accuracy, 89.39% and 72.81% in the PhysioNet dataset at the subject and group levels, respectively, demonstrating the complex feature learning ability of DL models. Due to its versatility, the F-FGCN model has a moderate error rate, signifying a high level of accuracy with only a 6.72% discrepancy.

**Table 3:** Performance comparisons on the PhysioNet dataset.

**Method**	**Based**	**Level**	**Num of subjects**	**Max accuracy**	**Avg accuracy**
FullyConvCNN	CNN	Group	20	73.94	65.17
		Subject	1	87.58	82.36
DenseCNN		Group	20	78.68	70.95
		Subject	1	94.71	87.83
ResCNN		Group	20	72.95	65.23
		Subject	1	91.53	85.97
LSTM-with-attention	RNN	Group	20	58.18	52.42
		Subject	1	81.27	74.64
BiLSTM-with-attention		Group	20	71.81	62.45
		Subject	1	74.52	81.91
RNN-with-attention		Group	20	67.31	50.78
		Subject	1	89.16	76.25
BiRNN-with-attention		Group	20	61.73	51.11
		Subject	1	85.62	78.58
Our Method	GCN	Group	105	58.29	51.77
			20	82.37	72.81
		Subject	1	96.11	89.39

In comparison to CNN-related methods like ResCNN and DenseCNN, which demonstrate high performances, our model exhibited comparable results. It achieved 82.37% top accuracy for a 20 participant group and 96.11% at the subject level. This underscores the efficacy of graph representation learning for EEG signal interpretation. Notably, at the group level, the accuracy between F-FGCN and the CNN models has a large difference, indicating a significant advantage in the predictive capabilities of F-FGCN, and establishing superiority in forecasting EEG tasks.

**Figure 8:**
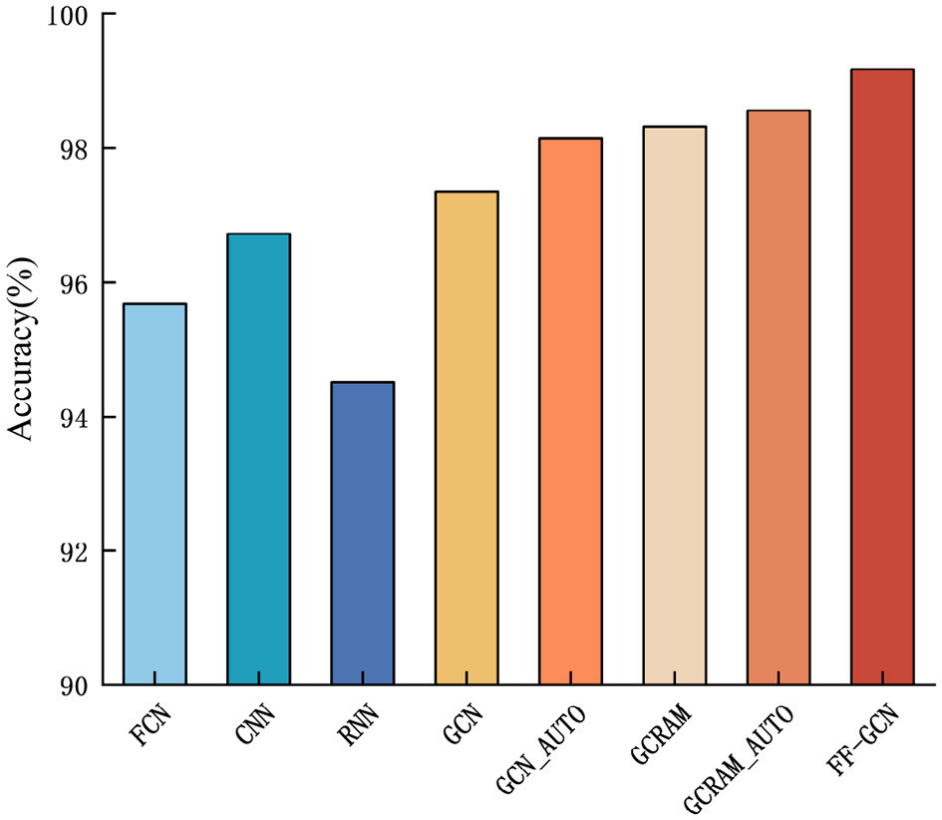
Comprehensive tests on BCI Competition III dataset.

To rigorously ascertain the generalizability and efficacy of our proposed algorithm, we have undertaken a series of comprehensive tests on BCI Competition III dataset. The outcome of these tests is graphically represented as Figure 8, clearly illustrating the performance benchmarks. It is noteworthy to mention that, as demonstrated in the accompanying figures, our algorithm has a clear accuracy advantage. This evidence indicates that our algorithm maintains a consistent and superior performance across various datasets, highlighting its potential for widespread application in the field.

Each experiment is conducted five times to derive the average accuracy rate. That is crucial to emphasize that our assessments were exclusively concerned with the quantity of graph convolutional layers, max pooling layers, and F-F layers. Model 7 demonstrated exceptional performance across all evaluated datasets.

As presented in Table 4, securing the count of graph convolutional layers at four enhances the effectiveness of the F-F mechanism in interpreting features. Escalating the number of said layers does not appreciably augment accuracy but extend the duration of the feature extraction phase. In parallel, the model yielded superior outcomes when utilizing a fixed quartet of F-F layers for feature classification.

**Table 4:** Performance comparisons of F-FGCN structures.

**Model**	**Pooling/Conv layers**	**Num of filters**	**GCN framework**	**F-F layers**	**F-F framework**	**Accuracy**
1	1	64	C-P	1	4	51.17%
2	1	64	C-P	2	64,4	54.73%
3	2	64,128	(C-P) × 2	2	64,4	55.42%
4	2	64,128	(C-P) × 2	3	64,32,4	58.65%
5	3	64,128,256	(C-P) × 3	3	256,128,4	65.49%
6	4	64,128,256,256	(C-P) × 4	3	256,128,4	69.34%
7	4	64,128,128,512	(C-P) × 4	4	512,256,128,4	72.81%
8	5	64,128,128,512	(C-P) × 4	5	512,256,128,64,4	72.57%
9	4	64,128,256,512,512	(C-P) × 5	4	512,256,128,4	72.32%
10	5	64,128,256,512,512	(C-P) × 5	5	512,256,128,64,4	72.78%

The FF-GCN adversarial training, which compares positive and negative samples to adjust weights, adds complexity and sensitivity to the training process due to its dual objective and dependence on the quality of negative samples. It increases the computational load as both sample types are processed, potentially slowing down each epoch.

## 5 Discussion

Our novel F-FGCN effectively decodes brain activity by leveraging the GCN model grounded in the F-F mechanism, thereby enhancing the accuracy of the algorithm.

F-FGCN manages to harmonize its performance across diverse subject data, thereby attaining extraordinary accuracy. When benchmarked against a multitude of alternative algorithms, our proposed model consistently demonstrates optimal classification performance. These results substantiate the assertion that the integration of topology and forward propagation in DL continues to exhibit formidable competitiveness in the MI-BCI decoding field.

Still, our study has certain limitations, particularly regarding the translation of electrode positioning and topology. Changes in electrode placements between different individuals can infuse distinct attributes in the features of the trained network. For different types of electrode locations used during an EEG examination, our network would need to be retrained to extract feature vectors effectively. Taking inspiration from related work (Hou et al., 2022b), we aim to explore methods to enhance versatility in future work.

## 6 Conclusion

In our study, we comprehensively investigate the task of MI EEG categorization, with consideration of brain network dynamics and neural signal transmission mechanism. We introduced the innovative F-FGCN model designed for four-class MI intents. F-FGCN amalgamates high-level individual interactions while considering EEG signal topology. By employing pre-training and F-F mechanism, our model further extracts feature vectors and amplifies the accuracy of the downstream classifier, resulting in the optimal detection results for the PhysioNet dataset. Our approach exhibited exemplary performance, cording the highest accuracy rates of 96.11% and 82.37% at the subject and group levels in the PhysioNet dataset, respectively.

In the future, we plan to integrate the F-F mechanism into our design of an end-to-end GCN network that could further enhance the accuracy of multi-classification tasks in MI. We also intend to explore the parallels between the human brain's signal propagation mechanism and the propagation process in DL. By efficiently leveraging EEG and label information, we aspire to apply this technology in areas such as humanoid robot control and the development of medical auxiliary equipment.

## Data availability statement

Publicly available datasets were analyzed in this study. This data can be found here: https://www.physionet.org/content/eegmmidb/1.0.0/.

## Author contributions

QX: Writing—original draft. YS: Writing—review & editing. HW: Writing—review & editing. YC: Writing—review & editing. HP: Writing—review & editing.
